# Low genetic diversity among historical and contemporary clinical isolates of felid herpesvirus 1

**DOI:** 10.1186/s12864-016-3050-2

**Published:** 2016-09-02

**Authors:** Paola K. Vaz, Natalie Job, Jacquelyn Horsington, Nino Ficorilli, Michael J. Studdert, Carol A. Hartley, James R. Gilkerson, Glenn F. Browning, Joanne M. Devlin

**Affiliations:** 1 Asia Pacific Centre for Animal Health, Faculty of Veterinary and Agricultural Sciences, The University of Melbourne, Parkville, VIC 3010 Australia; 2 Present address: Australian Animal Health Laboratory, CSIRO, 5 Portarlington Rd, East Geelong, VIC 3220 Australia; 3 Centre for Equine Infectious Diseases, Faculty of Veterinary and Agricultural Sciences, The University of Melbourne, Parkville, VIC 3010 Australia

**Keywords:** Felid herpesvirus, Genome, Recombination, Diversity, Feline

## Abstract

**Background:**

Felid herpesvirus 1 (FHV-1) causes upper respiratory tract diseases in cats worldwide, including nasal and ocular discharge, conjunctivitis and oral ulceration. The nature and severity of disease can vary between clinical cases. Genetic determinants of virulence are likely to contribute to differences in the in vivo phenotype of FHV-1 isolates, but to date there have been limited studies investigating FHV-1 genetic diversity. This study used next generation sequencing to compare the genomes of contemporary Australian clinical isolates of FHV-1, vaccine isolates and historical clinical isolates, including isolates that predated the introduction of live attenuated vaccines into Australia. Analysis of the genome sequences aimed to assess the level of genetic diversity, identify potential genetic markers that could influence the in vivo phenotype of the isolates and examine the sequences for evidence of recombination.

**Results:**

The full genome sequences of 26 isolates of FHV-1 were determined, including two vaccine isolates and 24 clinical isolates that were collected over a period of approximately 40 years. Analysis of the genome sequences revealed a remarkably low level of diversity (0.0–0.01 %) between the isolates. No potential genetic determinants of virulence were identified, but unique single nucleotide polymorphisms (SNPs) in the UL28 and UL44 genes were detected in the vaccine isolates that were not present in the clinical isolates. No evidence of FHV-1 recombination was detected using multiple methods of recombination detection, even though many of the isolates originated from cats housed in a shelter environment where high infective pressures were likely to exist. Evidence of displacement of dominant FHV-1 isolates with other (genetically distinct) FHV-1 isolates over time was observed amongst the isolates obtained from the shelter-housed animals.

**Conclusions:**

The results show that FHV-1 genomes are highly conserved. The lack of recombination detected in the FHV-1 genomes suggests that the risk of attenuated vaccines recombining to generate virulent field viruses is lower than has been suggested for some other herpesviruses. The SNPs detected only in the vaccine isolates offer the potential to develop PCR-based methods of differentiating vaccine and clinical isolates of FHV-1 in order to facilitate future epidemiological studies.

**Electronic supplementary material:**

The online version of this article (doi:10.1186/s12864-016-3050-2) contains supplementary material, which is available to authorized users.

## Background

Felid herpesvirus 1 (FHV-1), an alphaherpesvirus, causes upper respiratory tract disease in cats that is characterised by pyrexia, severe nasal and ocular discharge, conjunctivitis and oral ulceration. It is a common cause of necrotising upper respiratory tract infections (URI) in cats housed in shelters, with more than 50 % of cats shedding virus only one week after admission [1]. The virus typically replicates in epithelial cells of the conjunctiva and the upper respiratory tract and infects local neurons, establishing latency in the trigeminal, pterygopalatine and cranial cervical ganglia [2]. Although virus replication is generally limited to the upper respiratory tract and conjunctiva, viraemia has been detected during the acute phase of infection [3, 4] and a recent report has described a case of non-suppurative meningoencephalitis in cats, suggesting that FHV-1 has potential to cause more invasive disease [5]. Vaccination has been used to help control disease caused by FHV-1 since the mid-1970s. Both attenuated and inactivated vaccines are in use currently, and while they can reduce the severity of disease, they do not prevent infection [6–9].

Early studies demonstrated that isolates of FHV-1 were antigenically indistinguishable using conventional serological techniques. Additionally, a high level of genomic homogeneity was identified using restriction endonuclease cleavage analyses [10, 11]. A subsequent restriction endonuclease cleavage analysis study of 78 Japanese isolates detected three FHV-1 genotypes, although these genotypes still appeared to be very similar [12]. Since these early studies there has been only limited investigation of the extent of variation between vaccine and wild type strains of FHV-1.

This study aimed to use next generation sequencing to compare the genomes of contemporary Australian clinical isolates of FHV-1 to those of historical clinical isolates, including isolates that predate the introduction of live attenuated vaccines into Australia. Analysis of the genome sequences aimed to assess the level of genetic diversity, identify potential genetic markers that could influence the in vivo phenotype of the isolates and examine the sequences for evidence of recombination.

## Results

### Genome sequence analysis of 26 FHV-1 isolates

The full genome sequences of a panel of 26 FHV-1 isolates were assembled and analysed. This included 24 Australian clinical field isolates spanning nearly 40 years, as well as isolates from two currently available vaccines. The genome sequence of the prototype C-27 FHV-1 strain (GenBank:NC_013590), a historical isolate from North America, was also included in the analysis [13]. The genomes of the Feligen vaccine isolate of FHV-1 assembled by two different methods (*de novo* assembly and map to reference assembly) were identical, with the exception of the regions containing short sequence repeats. This provided a high level of confidence in genome assembly by mapping to a reference sequence and this method was used for all other FHV-1 isolates in this study. Genome sequence data have been deposited into GenBank under the accession numbers as listed in Table 1 [GenBank:KR296657 and GenBank:KR381779 - KR381803]. The size of the FHV-1 full genomes ranged from 133.5 kbp (isolate 3227–05) to 135.1 kbp (isolate 3225–05). Genome length variation occurred primarily as a result of differences in the large sequence repeat regions (IRS/TRS) and in the unique short region as a result of tandem repeat iterations.

**Table 1:** FHV-1 isolates sequenced in this study

Virus ID	Year	Origin^a^	Disease	Site	Vacc	Other^b^	GenBank	Reference
85/68	1968	Shelter A	URTI	Nasal swab	N	6 mo female, febrile	KR381787	[31]
117/68	1968	Shelter A	URTI	Nasal swab	N	7 wo	KR381780	[31]
124/68b	1968	Shelter A	URTI	Nasal swab	N	8 yo, female	KR381788	This study
135/68a	1968	Shelter A	URTI	Nasal swab	N	n/a	KR381786	This study
221/71	1971	Private	URTI, conjunct	Eye swab	N	3.5 wo female Persian	KR381781	This study
356/75b	1975	Blackburn	URTI	n/a	N	Siamese	KR381784	This study
384/75	1975	Mornington	Pneumonia	Lung	N	5 wo female Burmese	KR381782	This study
448/77	1977	Private	n/a	Pharynx swab	N	n/a	KR381783	This study
571/79	1979	Albert Park	Conjunct	Eye swab	N	Male	KR381785	This study
729/83	1983	Balwyn	Conjunct	n/a	Y	3 mo	KR381779	This study
3224/04	2004	Shelter B	URTI, oral ulcer	Nasal swab	Y	4 yo male DSH	KR381792	This study
3225/05	2005	Shelter B	Absent	Eye swab	Y	10 wo male DLH	KR381801	This study
3226/05	2005	Shelter B	URTI	Oral swab	Y	9 wo male DSH	KR381793	This study
3227/05	2005	Shelter B	Absent	Nasal swab	Y	15 wo male DSH	KR381802	This study
3228/05	2005	Shelter B	Oral ulcer	Nasal swab	Y	10 wo female DSH	KR381794	This study
3229/05	2005	Shelter B	URTI	Eye swab	n/a	16 wo male DLH	KR381795	This study
3230/05	2005	Shelter B	URTI	Nasal swab	Y	1 yo male DSH	KR381796	This study
3231/05	2005	Shelter B	Oral ulcer, conjunct	Oral swab	Y	8 wo male, DLH	KR381797	This study
3232/05	2005	Shelter B	Oral ulcer	Eye swab	Y	9 wo female DSH	KR381798	This study
3233/05	2005	Shelter B	URTI, diarrhea	Oral swab	Y	12 wo female DSH	KR381799	This study
3234/05	2005	Shelter B	URTI	Eye swab	Y	8 wo female DSH	KR381800	This study
3235/06	2006	Shelter B	URTI, conjunct	Oral swab	Y	5 wo male DSH	KR381789	This study
3236/06	2006	Shelter B	URTI	Eye swab	Y	4 yo male Siamese	KR381791	This study
3238/06	2006	Shelter B	URTI	Oral swab	Y	2 yo male DSH	KR381790	This study
3239/14	2013	USA	-	Vaccine	-	FeligenRCP Virbac	KR296657	-
3240/14	2013	USA	-	Vaccine	-	Companion Intervet	KR381803	-

^a^All isolates sequenced, excluding vaccine isolates, were from Victoria, Australia

^b^ *DSH* domestic short hair, *DLH* domestic long hair, *yo* year old, *mo* month old, *wo* week old, *URTI* upper respiratory tract infection, *Conjunct* conjunctivitis, *Y/N* yes/no, *n/a* data not available

**Fig. 1:**
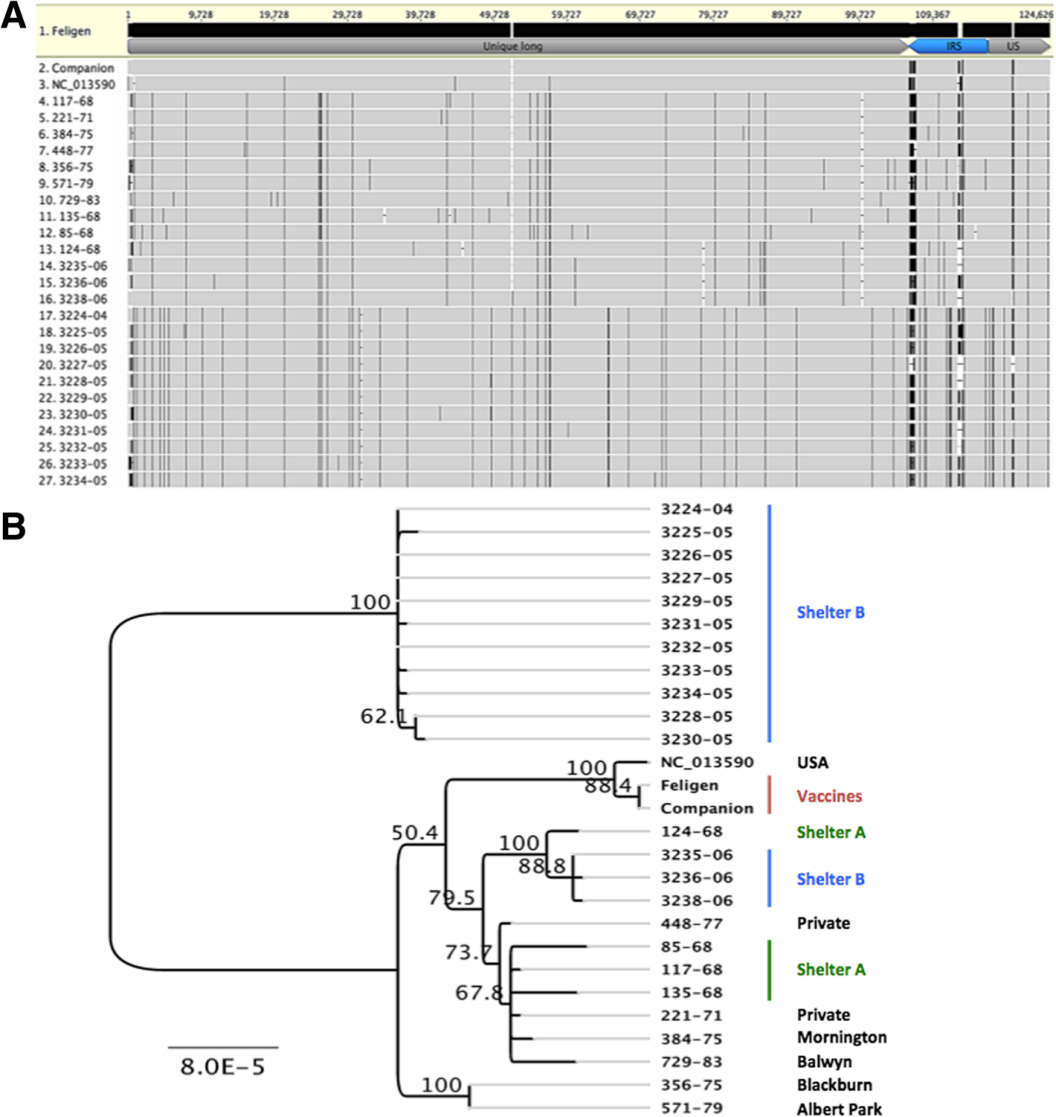
Nucleotide sequence alignment and phylogenetic tree of the complete genomes of 27 FHV-1 isolates, excluding the terminal repeat region. **a** Alignment of the complete genome sequences of FHV-1 isolates performed using MAFFT. The Feligen vaccine F2 isolate was used as the reference sequence. Vertical black lines indicate SNPs relative to the reference and dashes indicate sequence gaps. **b** Phylogenetic tree generated from the complete genome alignment, using the Jukes Cantor model and the neighbor-joining method. One thousand bootstrap replicates were used to assess the significance of the tree topology. The bar indicates nucleotide substitutions per site. The origin of isolates is indicated to the right of the isolate name

Despite the long time span over which the isolates were obtained, alignment of the FHV-1 genomes (Fig. 1a) revealed that diversity between them only ranged from 0.0 to 0.01 %. While the limited geographical range of many of the Australian isolates could have influenced this, three of the genomes analysed in this study were from isolates of North American origin. The most genetically diverse isolates (135/68 from 3225/05 and 3230/05), differed genome-wide by only 83 single nucleotide polymorphisms (SNPs), while some isolates shared 100 % nucleotide identity (excluding large regions of tandem repeat reiterations). The identical viruses included 356/75 with 571/79, while the eleven 2004/2005 field isolates differed by 0 to 4 SNPs, and the three 2006 isolates differed by 1 or 2 SNPs. The two vaccines were identical (excluding large reiterative repeat regions) and so are both likely to have been derived from the widely used F2 strain. The C-27 strain, a field strain isolated contemporaneously with the F2 vaccine strain from the USA, varied from the F2-derived vaccine strains by only 5 SNPs genome-wide (excluding large reiterative repeat regions) [14, 15]. The non-synonymous nucleotide differences (SNPs and insertions/deletions, or indels), as indicated by predicted amino acid residue differences between isolates, are provided in Table 2.

**Table 2:** Amino acid residue changes resulting from non-synonymous nucleotide polymorphisms, including insertions/deletions, compared to the reference isolate (F2 strain) of FHV-1

Gene	AA change^a^	Variant isolates ^b^
Unique long region		
UL1	F4L	135/68
UL3.5	V15I	2004/2005
UL5	T7I	124/68, 3235/06, 3236/06, 3238/06
UL13	S25P	85/68, 117/68, 124/68, 135/68, 221/71, 356/75, 385/75, 448/77, 571/79, 729/83,3235/06, 3236/06, 3238/06
UL16	F344V	2004/2005
UL17	R519Q	2004/2005
UL21	A48D	3234/05
UL25	T60A	2004/2005
UL27	P872L	3235/06, 3236/06, 3238/06
UL28	Q427P	All
UL29	S1089P	Field
UL30	M943I	3228/05, 3230/05
	I153T	729/83
UL35	R3S	NC_013590
UL36	G2503E	3230/05
	V1310M	124/68
	T2565M	221/71
	N2936-	135/68
	V2937-	135/68
	Q2980K	117/68
	P2838S	85/68, 117/68, 124/68, 135/68, 221/71, 356/75, 385/75, 448/77, 571/79, 729/83, 3235/06, 3236/06, 3238/06
UL37	L866F	2004/2005
UL38	I277M	Field
UL40	V66I	Field
UL44	E133K	All
UL45	R102H	729/83
UL49	T104I	3236/06
UL52	Y484C	Field
	S579I	3225/05
UL54	I206V	2004/2005
	S60P	135/68
UL55	I78M	Field
V1	D59G	124/68
ICP0	S412F	356/75, 571/79
Unique short region		
US7	M165T	Field
	P186S	124/68, 3235/06, 3236/06, 3238/06
Repeat region		
ICP4	T156N	124/68, 3235/06, 3236/06, 3238/06
	A419V	85/68, 117/68, 124/68, 135/68, 221/71, 3235/06, 3236/06, 3238/06
	H875Q	385/75
	V983A	356/75, 571/79, 3224/04, 3225/05, 3226/05, 3227/05, 3228/05, 3229/05, 3230/05, 3231/05, 3232/05, 3233/05, 3234/05
	E1034K	3235/06, 3236/06, 3238/06
	E1054G	2004/2005
US1	L261-	85/68

^a^ *AA* amino acid change

^b^Relative to the vaccine isolates (F2 FHV-1 strains from the Feligen/Companion vaccines). All = Variant found in all non-vaccine isolates, Field = Variant found only in all Australian isolates sequenced in this study, 2004/2005 = Variant unique to 2004/2005 field isolates

### Phylogenetic and recombination analyses of FHV-1 isolates

Phylogenetic analyses revealed that FHV-1 isolates fell into two main groups (Fig. 1b). Whilst some isolates with known geographical or temporal associations did cluster together (for example all 2004 and 2005 isolates), overall there was no clear relationship between viral genome sequences, the geographical location where the virus was isolated, the year of virus isolation or whether they predated the date of introduction of the attenuated FHV-1 vaccine into Australia in 1977. No clear association was detected between the genome sequence and the presence or nature of disease in the cat from which the virus was isolated.

## Discussion

The genome sequences of Australian isolates that predated the introduction of the FHV-1 vaccine into Australia (in 1977) had a high sequence identity with isolates obtained since then, and also with isolates originating from the USA. Overall, our data demonstrated that FHV-1 genomes are highly conserved, more so than any of the other herpesviruses analysed to date, with a similar study of equine herpesvirus 1 (EHV-1) isolates finding variation of between 5 and 173 SNPs between EHV-1 isolates [16]. Previous studies have suggested greater differences between FHV-1 isolates, including re-arrangements in the UL44 (gC) gene [17], restriction endonuclease cleavage site differences in the UL17 gene [18], and differences in *Mlu*I cleavage patterns across the genome [11]. However, all the isolates examined in our study had conserved gene arrangements, with only one SNP detected in UL44 (a non-synonymous substitution), and the predicted restriction endonuclease cleavage patterns generated from the sequence data varied mainly as a result of variations in short tandem repeat iterations.

This study included analyses of fourteen FHV-1 isolates from cats housed in an animal shelter between 2004 and 2006. The cluster of viruses from this shelter in 2004 and 2005 is of particular interest because of the apparent transience of a dominant virus in a shelter environment. The SNP profile of the viruses from 2004 to 2005 was distinct from those of all the other isolates sequenced, but this pattern was not detected in the 2006 isolates from the same shelter. The close phylogenetic relationship between the viruses isolated in 2004/2005, and between those collected in 2006, suggests endemic circulation in each of these periods, with clear evidence of displacement of the 2004/2005 viruses by those detected in 2006. This displacement may have occurred as a result of increased transmissibility of the newer viruses, or management changes, such as seasonal crowding [19], depopulation and repopulation of the shelter, or sampling bias (only samples free of feline calicivirus were selected for use in this study).

In vitro studies have reported a high rate of recombination in FHV-1 [20]. However, no evidence of recombination was detected between the 27 FHV-1 isolates, irrespective of genomic region analysed or the program used (*P* > 0.05). It is possible that recombination events may not have been detected because of the low levels of diversity among the viruses in our study. It is also possible that previous in vitro studies may have overestimated the likelihood of FHV-1 recombination because of the use of high multiplicities of infection and simultaneous infection, as both these factors have been shown in previous studies to increase the rate of recombination in vitro in other herpesviruses [21]. Recombination would be predicted in circumstances where the opportunity for co-infection is high and where the recombinant progeny have a survival advantage over the parent viruses and dominant field strains. While infective pressure in a shelter environment would be expected to allow concurrent infections with different FHV-1 isolates, there may be insufficient selective pressure on recombinants for them to emerge and persist in vivo in a natural (shelter) environment, particularly as the low levels of diversity among FHV-1 viruses may result in insufficient selective distinction for specific viruses to emerge in a population.

## Conclusions

Attenuated herpesvirus vaccines are used not only against FHV-1 in cats, but also against Marek’s disease virus and infectious laryngotracheitis virus (ILTV) in poultry, bovine herpesvirus 1 in cattle, pseudorabies virus in swine, and EHV-1 and equine herpes virus 4 (EHV-4) in horses. Previous studies of ILTV in poultry have demonstrated that attenuated vaccine strains can participate in natural recombination events that lead to the generation of more virulent progeny [22]. The risk of recombination involving other attenuated veterinary herpesvirus vaccines has not been evaluated comprehensively, but the risk appears to higher in some viruses (such as EHV-4) and lower in others (such as EHV-1) [16]. The results from this study suggest that the risk for FHV-1 vaccine recombination may also be low. Although the FHV-1 genome sequencing data from this study confirms the findings of earlier studies that FHV-1 s are relatively homogeneous [11] this study did detect SNPs unique to F2-derived vaccine strains, in genes UL28 and UL44. A search of all FHV-1 sequences available on GenBank identified only nine sequences covering these regions of the FHV-1 genomes, all relating to the UL44 gene. None of the sequences contained the vaccine-specific SNP, however the sequences were mostly of unknown origin apart from two; a BAC of the C-27 strain [GenBank:GM036023] and strain C7301 [GenBank:D86616]. There is the potential to use the F2-derived vaccine unique SNPs to differentiate vaccine FHV-1 isolates from other circulating FHV-1 s, and thus facilitate future epidemiological studies in FHV-1, especially to investigate the epidemiology of attenuated FHV-1 vaccines. Sequencing of more geographically diverse isolates will help confirm whether these SNPs are suitable markers for such studies.

## Methods

### Viruses and genome sequencing

Ten historical and fourteen recent Australian FHV-1 isolates, as well as two live attenuated vaccines currently available in Australia (Table 1) were selected from our laboratory archive. Historical isolates were obtained from clinical samples submitted to our laboratory as a component of past diagnostic services. Recent samples were collected from shelter-housed cats in 2004 to 2006 with approval from the Faculty of Veterinary Science, Animal Ethics Committee at The University of Melbourne (reference number 0004055.1) and with the consent from the shelter owners. Viruses were propagated in cultures of Crandell Rees feline kidney (CRFK) cells [23] for six passages, during which three plaque purifications were performed. CRFK cells are derived from a normal, female, 12 weeks old cat and are commercially available (ATCC CCL-94). Isolates selected for sequencing dating from 2004 to 2006 were all from animals that were negative for feline calicivirus, as determined by RT-PCR [24]. Viral nucleocapsid genomic DNA was purified and sequenced as described previously [16]. Libraries were prepared using 50 ng of viral genomic DNA with the Illumina Nextera DNA library preparation kits according to manufacturer’s instructions, and loaded onto an Illumina MiSeq. Sequencing was carried out using a 300 cycle V2 SBS kit (Illumina) in paired-end format. Reads were trimmed to an error probability limit of 0.5 % and mapped against the prototype FHV-1 sequence, strain C-27 [GenBank:NC_013590], using medium-low sensitivity options in the bioinformatics package Geneious V6.1.7 [25]. For comparison, a *de novo* assembly of the genome of the F2 strain of FHV-1 from the Feligen vaccine (Virbac) was also performed using the medium-low sensitivity settings in Geneious. The details of the sequencing metrics are provided in Additional file 1: Table S1.

### Phylogenetic and recombination analyses

Alignments of the complete genome sequences of the FHV-1 isolates, excluding the terminal repeat regions (TRS), were prepared using the Multiple Alignment with Fast Fourier Transformation (MAFFT) version 7 plugin in Geneious [26]. The F2 vaccine strain of FHV-1 was used as the reference sequence. The historical C-27 strain of FHV-1 was also included throughout the analyses. Phylogenetic analyses were performed on the complete genome sequences, excluding the TRS and large sequence gaps, using the neighbor-joining method in Geneious with the Jukes Cantor model of nucleotide substitution [27]. One thousand bootstrap replicates were used to assess the significance of the phylogenetic tree topology.

To detect evidence of historical recombination events between the isolates, two programs were used, SplitsTree4 [28] and RDP4 [29], as previously described [16]. Short tandem repeat regions were identified in the aligned genomes prior to analysis using the Phobos plugin in Geneious V6.1.7. Default settings and score constraints for satellites with a maximum repeat unit length of 50 nucleotides were used. The identified repeat regions were removed from all viruses in the alignment as the methods used in this study do not allow the length of these regions to be accurately determined, and differences in the length of these regions could give rise to false positive recombination events being detected. The resultant whole genome alignments, as well as the separate UL, US and IRS genomic region alignments, were analysed for evidence of recombination. Splits network trees were generated with an uncorrected P characters transformation model, ignoring gapped sites. Other models were tested but yielded no significant topological differences. Statistical analyses of the recombination networks were performed using the Phi test as implemented in SplitsTree4 [30]. In RDP4 six different methods were used to assess the sequences for recombination breakpoints, MaxChi, Bootscan, SiScan, Chimaera, GENECONV and RDP, with default RDP4 settings used throughout.

## Additional file

Additional file 1: Table S1. FHV-1 sequencing metrics, viral genome sequence details. (DOCX 17 kb)

## Abbreviations

FHV-1: Felid herpesvirus 1
ILTV: Infectious laryngotracheitis virus
EHV: Equine herpesvirus
UL: Unique long
US: Unique short
IRS/TRS: Internal/external repeat short
SNP: Single nucleotide polymorphism
CRFK: Crandell Rees feline kidney cells
PCR: Polymerase chain reaction
RDP4: Recombination detection program version 4
