# Supplementary material for: Low genetic diversity among historical and contemporary clinical isolates of felid herpesvirus 1
Source: BMC Genomics. 2016 Sep 2;17(1):704. doi: 10.1186/s12864-016-3050-2 (PMC5010698; doi:10.1186/s12864-016-3050-2)
Supplement: Additional file 1: Table S1. — FHV-1 sequencing metrics, viral genome sequence details. (DOCX 17 kb) [file 12864_2016_3050_MOESM1_ESM.docx]

Additional file 1: Table S1. FHV-1 sequencing metrics

|  | **FHV-1 isolates** | | | | | | | | | | | | | | | | | | | | | | | | | |
| --- | --- | --- | --- | --- | --- | --- | --- | --- | --- | --- | --- | --- | --- | --- | --- | --- | --- | --- | --- | --- | --- | --- | --- | --- | --- | --- |
|  | **85-68** | **117-68** | **124-68b** | **135-68a** | **221-71** | **356-75b** | **384-75** | **448-77** | **571-79** | **729-83** | **3224-04** | **3225-05** | **3226-05** | **3227-05** | **3228-05** | **3229-05** | **3230-05** | **3231-05** | **3232-05** | **3233-05** | **3234-05** | **3235-06** | **3236-06** | **3238-06** | **Feligen** | **Comp** |
| **Total reads (millions)** | 1.76 | 1.55 | 2.31 | 1.43 | 2.01 | 1.55 | 2.06 | 1.27 | 1.81 | 2.19 | 1.40 | 2.13 | 2.05 | 0.11 | 0.62 | 0.83 | 0.85 | 0.66 | 1.80 | 0.54 | 1.83 | 0.36 | 2.02 | 0.37 | 0.48 | 2.07 |
| **Mapped reads (millions)** | 1.46 | 1.26 | 2.00 | 1.18 | 1.90 | 1.37 | 1.91 | 1.22 | 1.61 | 1.52 | 1.34 | 1.78 | 1.50 | 0.09 | 0.57 | 0.73 | 0.80 | 0.42 | 1.38 | 0.49 | 1.72 | 0.32 | 1.87 | 0.34 | 0.43 | 1.54 |
| **Breadth of coverage (%)*** | 100 | 100 | 100 | 100 | 100 | 100 | 100 | 100 | 100 | 100 | 99.6 | 100 | 100 | 99.9 | 99.8 | 99.9 | 99.9 | 99.8 | 100 | 99.9 | 100 | 99.9 | 100 | 100 | 99.9 | 100 |
| **Av read length (bp)** | 249 | 248 | 248 | 249 | 249 | 248 | 249 | 249 | 249 | 248 | 147 | 248 | 249 | 149 | 194 | 186 | 194 | 182 | 250 | 192 | 249 | 171 | 250 | 203 | 195 | 249 |
| **Mean depth of coverage ^** | 2695 | 2325 | 3675 | 2184 | 3510 | 2525 | 3535 | 2235 | 2975 | 2802 | 1466 | 3257 | 2770 | 105 | 827 | 1011 | 1158 | 568 | 2564 | 707 | 3176 | 408 | 3470 | 514 | 631 | 2843 |
| **Genome size (kb)** | 134.9 | 134.8 | 134.7 | 134.8 | 135.0 | 134.4 | 134.7 | 135.1 | 134.6 | 135.0 | 134.6 | 135.1 | 134.6 | 133.5 | 134.1 | 134.3 | 134.3 | 134.1 | 134.5 | 134.2 | 134.7 | 134.3 | 134.8 | 134.2 | 134.5 | 135.0 |

^*^Breadth of genome coverage at a minimum depth of 1 read

^^^Number of mapped reads **×** average read length **/** total length of the genome
